# The use of prophylactic transcatheter arterial embolization in Forrest type II ulcer patients without attempted endoscopic hemostasis

**DOI:** 10.1097/MD.0000000000047890

**Published:** 2026-03-06

**Authors:** Xiyue Zhang, Dongqing Wang, Qingliang Zhu, Hailong Zhang

**Affiliations:** a School of Clinical Medical Sciences, Southwest Medical University, Luzhou, China; b Department of Gastroenterology, Southwest Medical University, Luzhou, China.

**Keywords:** peptic ulcer, transcatheter arterial embolization, upper gastrointestinal bleeding

## Abstract

This study aims to report the utility of prophylactic transcatheter arterial embolization (TAE) in Forrest type II ulcer patients without attempted endoscopic hemostasis and evaluate the factors associated with positive angiography. Forrest type II ulcer patients without attempted endoscopic hemostasis underwent prophylactic TAE between September 2019 and July 2023 at a single academic medical center were retrospectively reviewed. Patient demographics, clinical presentations, comorbidities, medications, endoscopy findings, laboratory investigations, the time intervals between endoscopy and TAE, interventional procedure findings, the occurrences of rebleeding, complications and clinical outcomes were recorded. The outcome measures were technical and clinical success, procedure related complications, recurrent bleeding and 30-day mortality. The factors associated with positive angiography were also assessed with univariate analysis and multivariate analysis. 12 patients (36.4%) had gastric ulcers and 21 (63.6%) had duodenal ulcers. 22 patients (66.7%) presented with Forrest IIa ulcers and 11 (33.3%) presented with Forrest IIb ulcers. Technical success rate was 100% without TAE related complication. Clinical success rate was 97.0% and rebleeding rate was 3.0%. 15 patients (45.5%) had positive angiography. Of them, 9 had contrast extravasation and 6 had pseudoaneurysms. Univariate analysis of the factors associated with positive angiography showed differences in sex (93.3% vs 61.1%, respectively, *P* = .046) and history of NSAIDs (73.3% vs 22.2%, respectively, *P* = .005). Multivariate analysis associated with positive angiography showed differences in history of NSAIDs (OR = 8.76, 95 CI = 1.61–47.62, *P* = .012). Prophylactic TAE is a safe, and effective method for treating Forrest type II ulcer patients without attempted endoscopic hemostasis.

## 1. Introduction

Peptic ulcer bleeding is the leading cause of acute nonvariceal upper gastrointestinal bleeding, responsible for approximately 31% to 67% of all cases.^[1]^ Despite medical and endoscopic advances in managing ulcer bleeding, rebleeding still occurs in 10% to 30% of patients, mortality in patients with further bleeding increases by approximately threefold.^[2,3]^ At this point, preventing rebleeding is crucial in patients with high-risk peptic ulcer.

The Forrest classification is a clinically useful tool to identify patients who are at high risk of rebleeding, it differentiates ulcers with a spurting hemorrhage (Forrest Ia), an oozing hemorrhage (Forrest Ib), with a visible vessel (Forrest IIa), an adherent clot (Forrest IIb), hematin on the ulcer base (Forrest IIc), and a clean ulcer base (Forrest III). Endoscopic therapy is indicated in Forrest type Ia, Ib, IIa, IIb ulcers since these ulcers have high rebleeding rates.^[4,5]^ However, Forrest type IIa and IIb are recent bleeding stigmata, some gastroenterologists are reluctant to aggressive endoscopic treatment since this may lead to massive bleeding and perforation. Meanwhile, endoscopic therapy still faces a great challenge in patients with active bleeding ulcers, large ulcers (size >1.5 cm), and ulcers with large-sized nonbleeding vessels (diameter >2 mm), who have a high risk of rebleeding after endoscopic therapy.^[6,7]^ Endoscopic treatment of high-risk patients with Forrest type IIb ulcers is also controversial.^[8]^ Based on the above-mentioned reasons, only few Forrest type II ulcer patients receive endoscopic treatment in our unit.

Transcatheter arterial embolization (TAE) was first introduced by Rösch and colleagues in 1972, and since then, was used as an effective and safe method in cases of refractory and rebleeding increasingly.^[9,10]^ Recently, several studies found that prophylactic TAE followed primary endoscopic therapy may reduce rebleeding rate in patients with high-risk bleeding peptic ulcer including Forrest type II ulcers.^[11]^ It is not clear which 1 is more effective, arterial embolization alone or endoscopic treatment alone. There is also no data evaluating TAE alone as a preventive tool to reduce the rebleeding rate in high-risk bleeding peptic ulcer. We hypothesized that TAE alone in patients with high-risk ulcers may reduce recurrent bleeding and improves their outcomes. Meanwhile. In our unit, Forrest type IIa and IIb are recent bleeding stigmata, gastroenterologists prefer to choose TAE to prevent rebleeding rather than aggressive endoscopic treatment since this may lead to massive bleeding and perforation. Hence, we reported our experiences and aimed to evaluate the safety and efficacy of prophylactic TAE in Forrest type II ulcer patients without attempted endoscopic hemostasis, the factors associated with positive angiography were also analyzed.

## 2. Materials and methods

### 2.1. Patient selection

**Figure 1.**
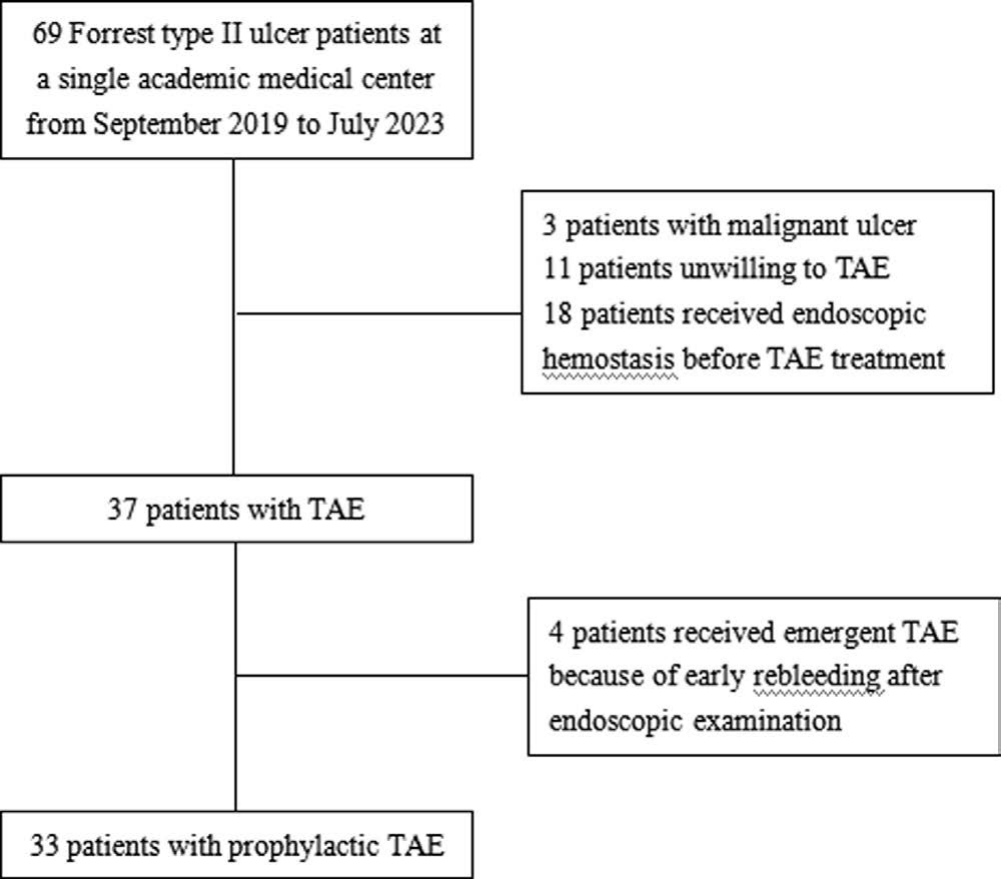
Flow diagram of patient enrollment.

This is a retrospective, monocentric study, investigating consecutive patients who underwent prophylactic TAE for preventing rebleeding related to Forrest type II peptic ulcer without attempted endoscopic hemostasis between September 2019 and July 2023 (Fig. 1). Patients were excluded if one or more following criteria were met: unwilling or had contraindication to TAE; received endoscopic hemostasis before prophylactic TAE; received emergent TAE because of early rebleeding; malignant ulcer; incomplete data. Written informed consent from patients was waived by Clinical Trial and Biomedical Ethics Committee of The Affiliated Hospital of Southwest Medical University due to the retrospective nature of this study. All procedures of this work were carried out in accordance with the guidelines and regulations of the institutional and national ethics committee.

### 2.2. Data collection and assessments

The medical records of patients who underwent a prophylactic TAE in The Affiliated Hospital of Southwest Medical University between September 2019 and July 2023, were scrutinized retrospectively. Data retrieved included patient demographics, clinical presentations, comorbidities, medications, endoscopy findings, laboratory investigations, the time intervals between endoscopy and TAE, interventional procedure findings, the occurrences of rebleeding, complications and clinical outcomes. The Rockall score was calculated and estimates the risk of patients after acute upper gastrointestinal bleeding for rebleeding. The Forrest classification was used to grade the severity of the ulcers.

### 2.3. Outcomes and definitions

The primary outcome of this study was the rate of 30-day rebleeding. Rebleeding was defined as clinically or endoscopically externalized bleeding and/or a bleeding with a >2.0 g/dL decrease in the hemoglobin level after TAE. We followed patients for 30 days after TAE. We contacted those by phone on day 30 if they were discharged earlier.

The second outcomes included technical and clinical success, 30-day and in-hospital mortality; the factors associated with positive angiography. Technical success was defined as absence of contrast extravasation at the end of the angiographic embolization procedure including occlusion of the bleeding vessel or exclusion of a pseudoaneurysm, or complete embolization of left gastric and/or right gastric artery and/or gastroepiploic artery for gastric ulcer depending on ulcer location, gastroduodenal artery (GDA) and/or right gastric artery for duodenal ulcer in the absence of active contrast extravasation or a pseudoaneurysm when empiric embolization was performed. Clinical success was defined as no clinical or laboratory signs of rebleeding within 30 days after TAE. Positive angiography was defined as active contrast extravasation and/or pseudoaneurysm.

### 2.4. Technique

Patients received endoscopy as the initial examination for gastrointestinal bleeding, marking clips were placed near the ulcer. All angiographic embolization procedures were performed by experienced interventional radiologists under local anesthesia. After gaining patient’s informed consent, percutaneous access to the right common femoral artery was made using a 5F sheath. A 5-Fr angiographic catheter was inserted into femoral artery, and thereafter celiac and superior mesenteric angiograms were performed. If no culprit artery was detected, superselective catheterization using microcatheter of the GDA and/or right gastric artery for duodenal ulcer, left gastric and/or right gastric artery and/or gastroepiploic artery for gastric ulcer depending on ulcer location were performed to identify if the artery is bleeding. If routine angiography did not show any bleeding focus, the catheter tip was positioned in branches closest to hemoclips to identify if the branch is bleeding. Embolization of the culprit artery was performed using micro-coils, gelfoam, glue. Embolic materials were used mixed or alone based at the culprit artery and the discretion of the attending interventional radiologist. Empiric embolization of the GDA and/or right gastric artery for duodenal ulcer, left gastric and/or right gastric artery and/or gastroepiploic artery for gastric ulcer depending on ulcer location or guided by hemoclips was performed in the absence of positive angiography. The complete occlusion of the target vessel or occlusion of the vessels as near as possible to the extravasation or marking clip was considered as the endpoint.

### 2.5. Statistical analyses

Continuous variables, expressed as a median and interquartile range, were analyzed using Student *t* test or nonparametric tests (Mann–Whitney test) for those not following a normal distribution. Categorical variables were presented as numbers and frequencies. Differences between categorical variables were analyzed using Pearson chi-squared test or Fisher exact probability test as appropriate. Variables associated with positive angiography with *P* <.1 in univariate analysis were included in multivariate analysis, using multivariable logistic regression. The significance level was set at *P*<.05, and the 2-sided P values were reported. The SPSS 22.0 statistical package (SPSS Inc., Chicago) was used for data analysis.

## 3. Results

### 3.1. Baseline characteristics of included patients

A total of 33 consecutive patients (75.8% males; mean age 64 years ± 14 years; range 18–91 years) were included between September 2019 and July 2023 in this study. The Demographic characteristics, history of ulcer disease, medications, Endoscopic findings are listed in Table 1. 11 patients (33.4%) had the history of peptic ulcer or peptic ulcer bleeding. 15 patients (45.5%) had the history of taking nonsteroidal anti-inflammatory drugs (NSAIDs) and 1 patient (3.0%) had the history of taking warfarin. 12 patients (36.4%) had gastric ulcers and 21 (63.6%) had duodenal ulcers. 22 patients (66.7%) presented with Forrest IIa ulcers and 11 (33.3%) presented with Forrest IIb ulcers. The time interval between endoscopy and TAE was 15.5h ± 18.0h (2h-64h).

**Table 1:** Baseline characteristics of patients.

Demographic characteristics	Male	25/33 (75.8%)
	Age (year: mean, SD, range)	64 ± 13.9 (18–91)
	Hemoglobin (g/L: mean, SD, range)	69.5 ± 20.6 (32–128)
	Hematocrit (L/L: mean, SD, range)	0.21 ± 0.05 (0.11–0.35)
	Platelet count (10^3^/mm^3^: mean, SD, range)	210.4 ± 114.2 (65–669)
	Prothrombin time (s: mean, SD, range)	14.9 ± 2.4 (11.0–22.4)
	INR (mean, SD, range)	1.2 ± 0.23 (0.94–1.93)
	Activated partial thromboplastin time (s: mean, SD, range)	32.4 ± 6.51 (20.2–43.6)
	SBP (mm Hg: mean, SD, range)	116 ± 22 (75–155)
	Transfusion (quantity of units: mean, SD, range)	5.4 ± 4.2 (0–17.5)
Previous ulcer disease	History of peptic ulcer	6/33 (18.2%)
	History of bleeding peptic ulcer	5/33 (15.2%)
Medications	NSAIDs	15/33 (45.5%)
	Warfarin	1/33 (3.0%)
Endoscopic findings	Gastric ulcer	12/33 (36.4%)
	Body	4/12 (33.3%)
	Antrum	7/12 (58.3%)
	Pylorus	1/12 (8.3%)
	Duodenal ulcer	21/33 (63.6%)
	Bulb	15/21 (71.4%)
	Descending	6/21 (28.6%)
	Ulcer size (cm: mean, SD, range)	3.0 ± 1.5 (0.4–3.0)
Forrest classification	IIa	22/33 (66.7%)
	Gastric ulcer	8/22 (36.4%)
	Duodenal ulcer	14/22 (63.6%)
	IIb	11/33 (33.3%)
	Gastric ulcer	4/11 (36.4%)
	Duodenal ulcer	7/11 (63.6%)
Interval time between endoscopy and TAE	Overall (hour: mean, SD, range)	15.5 ± 18.0 (2–64)
	Gastric ulcer	13.3 ± 16.0 (2–48)
	Duodenal ulcer	16.8 ± 19.4 (2–64)

INR = International normalized ratio, NSAIDs = nonsteroidal anti-inflammatory drugs, SBP = systolic blood pressure, SD = standard deviation, TAE = transcatheter arterial embolization.

### 3.2. Angiographic and interventional outcomes

TAE was performed under local anesthesia in all patients. Technical success rate was 100% without TAE related complication. 15 patients (45.5%) had positive angiography (Table 2). Of them, 9 had contrast extravasation and 6 had pseudoaneurysms. Left gastric artery, right gastric artery and gastroepiploic artery were identified as culprit vessels in 2 patients (25.0%), 3 patients (37.5%) and 3 patients (37.5%) with gastric ulcers bleeding respectively. Right gastric artery and gastroduodenal artery were identified as culprit vessels in 3 patients (42.9%) and 4 patients (57.1%) with duodenal ulcer bleeding respectively. 18 patients had negative angiography and received empiric embolization according to the location of ulcers and marked hemoclips. Coils combined with gelfoam (60.6%) were the most used embolic agents, other agents included coils alone (3.0%), coils combined with cyanoacrylate (6.1%), coils combined with gelfoam and cyanoacrylate (21.2%), cyanoacrylate alone (9.1%).

**Table 2:** Angiographic and interventional outcomes.

Angiographic findings	Positive angiography	15/33 (45.5%)
	Contrast extravasation	9/33 (27.3)
	Pseudoaneurysm	6/33 (18.2)
	Negative angiography	18/33 (54.5)
Gastric ulcer	Culprit vessels	8/15 (53.3)
	Left gastric artery	2/8 (25.0)
	Right gastric artery	3/8 (37.5)
	Gastroepiploic artery	3/8 (37.5)
Duodenal ulcer	Culprit vessels	7/15 (46.7)
	Right gastric artery	3/7 (42.9)
	Gastroduodenal artery	4/7 (57.1)
Embolic agents	Coils	1/33 (3.0)
	Coils + gelfoam	20/33 (60.6)
	Coils + cyanoacrylate	2/33 (6.1)
	Coils + gelfoam + cyanoacrylate	7/33 (21.2)
	Cyanoacrylate	3/33 (9.1)
Technical success	–	100
Clinical success	Overall	97.0
	Gastric ulcer	11/12 (91.7)
	Duodenal ulcer	21/21 (100)
Rebleeding	Overall	1/33 (3.0)
	Gastric ulcer	1/12 (8.3)
	Duodenal ulcer	0/21 (0)
Major complications	–	None
30-day mortality	Overall	1/33 (3.0)
	Rebleeding	1/33 (3.0)
	Others	0 (0)
Survival	Overall	97.0
	Gastric ulcer	11/12 (91.7)
	Duodenal ulcer	21/21 (100)

Clinical success rate was 97.0% and rebleeding rate was 3.0%. Rebleeding and mortality occurred in 1 patient (3.0%) with Forrest IIa gastric ulcer 5 days after empiric TAE. The patient was in shock and unconscious soon after rebleeding, his family refused further treatment and the patient died 6 hours later.

### 3.3. Factors associated with positive angiography

A univariate analysis of the factors associated with positive angiography showed differences in sex (93.3% vs 61.1%, respectively, *P* = .046) and history of NSAIDs (73.3% vs 22.2%, respectively, *P* = .005). Multivariate analysis associated with positive angiography showed differences in history of NSAIDs (Table 3). The probability of positive angiography in patients with history of NSAIDs was 8.76 times higher than in those without history of NSAIDs (OR = 8.76, 95CI = 1.61–47.62, *P* = .012).

**Table 3:** Factors associated with positive angiography.

	Positive angiography (n = 15)	Negative angiography (n = 18)	*P*	OR (95% CI)	*P* ^'^
Age, year	61.7 ± 15.3	66.1 ± 12.8	.37	–	–
Sex, male, n (%)	14 (93.3%)	11 (61.1%)	.046	0.13 (0.01–1.50)	.103
SBP, mm Hg	111.00 ± 22.03	119.11 ± 21.75	.27	–	–
Hemoglobin (Hb), g/dL	64.00 ± 17.80	74.17 ± 22.06	.16	–	–
Hematocrit, L/L	0.20 ± 0.05	0.23 ± 0.06	.18	–	–
Platelet count, 10^3^/mm^3^	238.73 ± 147.82	186.83 ± 72.56	.20	–	–
Prothrombin time, s	15.01 ± 1.82	14.77 ± 2.88	.78	–	–
INR	1.19 ± 0.16	1.16 ± 0.28	.67	–	–
Activated partial thromboplastin time, s	33.31 ± 6.28	31.56 ± 6.76	.45	–	–
Transfusion	6.50 ± 5.01	4.56 ± 3.34	.19	–	–
Rock	2.93 ± 0.96	3.11 ± 0.96	.60	–	–
History of peptic ulcer (yes/no)	8/7	14/4	.16	–	–
NSAIDs (yes/no)	11/4	4/14	.005	8.76 (1.61–47.62)	0.012
Location of ulcers (gastric/duodenal)	8/7	4/14	.163	–	–
Ulcer size	1.55 ± 0.89	1.49 ± 0.90	.87	–	–
Forrest classification (IIa/IIb)	8/7	14/4	.27	–	–
Interval time between endoscopy and TAE	11.02 ± 13.77	19.25 ± 20.59	.20	–	–

CI = confidence, INR = International normalized ratio, NSAIDs = nonsteroidal anti-inflammatory drugs, OR = odds ratio, SBP = systolic blood pressure, SE = standard error, TAE = transcatheter arterial embolization.

## 4. Discussion

In our study, prophylactic TAE in Forrest type II ulcer patients without aggressive endoscopic hemostasis is safe and effective with a high technical success rate (100%) and low rebleeding rate (3.0%), no TAE related complication occurred. The low rebleeding rate is comparable with endoscopic treatment ranging from 1.1% to 20.8%, but indicate a substantial improvement when compared with proton-pump inhibitors alone ranging from 11.6% to 22.9 %.^[12,13]^ The technical success rate is comparable with other studies ranging from 94% to 100%.^[14–17]^ One patient with Forrest type IIa ulcer located in the posterior wall of gastric body died of rebleeding after empirical embolization of left and gastric artery with gelatin sponge particles and coils, gastroduodenal artery with gelatin sponge particles.

Data about angiographic results of Forrest type II ulcer are lacking. However, despite no active bleeding during endoscopy, we were surprised to find that 45.5% of patients have positive angiography, including 27.3% of patients had direct contrast extravasation and 18.2% had pseudoaneurysms. This result is similar to a previous study reported by Song et. al in which 44% of patients with bleeding peptic ulcers presented with large clots, large exposed vessels or large ulcerative lesions had direct contrast extravasation on the superselective angiography guided by hemoclips.^[18]^ Several reasons may explain these results. First, Forrest type II ulcer is prone to rebleed, previous studies revealed that rebleeding rates range from 30% to 50% in Forrest type II ulcer patients without aggressive endoscopic hemostasis.^[13]^ Second, theoretically, contrast agent injection causes increase in intravascular pressure, culprit vessels rupture again before their laceration heal completely. Third, superselection of a distally smaller artery with a microcatheter guided by marked hemoclips placed via gastroscopy results in a higher positive rate.^[18]^

Computed tomography angiography (CTA) is a useful diagnostic tool for evaluating the presence and location of gastrointestinal bleeding due to its noninvasiveness, speed, and sensitivity.^[19]^ It is generally recommended as a routine examination for active gastrointestinal bleeding or obscure gastrointestinal bleeding.^[20]^ The potentially promising algorithm to consider is that patients should immediately undergo angiography when contrast extravasation or pseudoaneurysms are found on CTA. However, we didn’t routinely perform CTA for Forrest type II ulcers, because Forrest type IIa and IIb ulcers are recent bleeding stigmata, not active bleeding, contrast extravasation may not be found by CTA if the bleeding is intermittent. Another important reason is the presence of marked hemoclips, which can interfere with the interpretation of the examination.

History of NSAIDs was a factor associated with positive angiography in this study, the reason is not clear. There are some evidences suggesting that NSAIDs can cause platelet dysfunction and prolonging the bleeding time,^[21,22]^ this may play a role in higher rate of positive angiography among NSAIDs users. Although no studies have explored whether depth of ulceration, blood vessel involvement is different in NSAIDs users and nonusers, some studies have found NSAIDs associate with larger peptic ulcer and peptic ulcer perforation,^[23]^ these may indicate more serious injury from NSAIDs in the development of PPU. However, more samples are needed to avoid concerns about imprecision due to small samples, wide confidence intervals leading to lack of precision in the effect estimates.

The adequate time when the patients with Forrest type II ulcer should receive TAE remains unclear. Most rebleeding reported occurs within the first 72 hours,^[24,25]^ endoscopic follow-up of Forrest Ia to IIb ulcers showed healing with a clean base by day 3 to 4.^[26–28]^ Based on the above-mentioned reasons, receiving TAE within 72 hours appears to be more appropriate. In our study, all patients received TAE within 72 hours, the maximum interval between endoscopy and TAE was 56 hours and the minimum was 2 hours. Theoretically, the shorter the interval, the more likely the angiography is positive. Unfortunately, univariate analysis and multivariate analysis of the factors associated with positive angiography failed to show statistically significant differences in terms of interval time between endoscopy and TAE. This may be explained by the fact that bleeding is intermittent, and our limited sample size.

The present study has several limitations. First, this is a single-center, retrospective study with a nonrandomized, noncontrolled design limiting its generalizability. The noncontrolled design was greatly attributed to the fact that endoscopic treatment is not the prevailing treatment choice for Forrest type II ulcer in our unit and only few Forrest type II ulcer patients did not receive prophylactic TAE, the ulcers treated with endoscopy or conservative therapy were also smaller in diameter than those treated with TAE. Second, the sample size of this study is small, further subanalyses are infeasible, including gastric or duodenal ulcer, Forrest IIa or IIb, different ulcer sizes and so on. Nevertheless, the results of this study are noteworthy, and suggest that a larger randomized trial would be of value to investigate the utility of prophylactic TAE in comparison with endoscopic or conservative treatment.

In conclusion, this study demonstrates that prophylactic TAE in Forrest type II ulcer patients without attempted endoscopic hemostasis may be safe, and effective with minimal rebleeding rate. It may have potential to be an alternative treatment to prevent rebleeding in Forrest type II ulcer patients without attempted endoscopic hemostasis. Further studies are needed in order to finally confirm the effect and safety of TAE, especially compared with endoscopic or conservative treatment.

## Author contributions

**Conceptualization:** Xiyue Zhang, Dongqing Wang, Hailong Zhang.

**Data curation:** Xiyue Zhang.

**Formal analysis:** Xiyue Zhang, Dongqing Wang, Qingliang Zhu.

**Investigation:** Xiyue Zhang, Dongqing Wang, Qingliang Zhu, Hailong Zhang.

**Methodology:** Xiyue Zhang, Hailong Zhang.

**Project administration:** Qingliang Zhu.

**Resources:** Dongqing Wang.

**Software:** Xiyue Zhang, Dongqing Wang.

**Supervision:** Hailong Zhang.

**Validation:** Hailong Zhang.

**Visualization:** Hailong Zhang.

**Writing – original draft:** Xiyue Zhang, Dongqing Wang, Qingliang Zhu.

**Writing – review & editing:** Hailong Zhang.
